# Magnetite/Poly(ortho-anisidine) Composite Particles and Their Electrorheological Response

**DOI:** 10.3390/ma14112900

**Published:** 2021-05-28

**Authors:** Qi Lu, Jin-Hee Lee, Jin Hyun Lee, Hyoung Jin Choi

**Affiliations:** 1 Department of Polymer Science and Engineering, Inha University, Incheon 22212, Korea; 22192255@inha.edu (Q.L.); wlsgml8124@naver.com (J.-H.L.); 2 Program of Environmental and Polymer Engineering, Inha University, Incheon 22212, Korea; 3 Polymer Research Center, Inha University, Incheon 22212, Korea

**Keywords:** magnetite, poly(o-anisidine), core–shell, electro-magneto-rheological, electrorheology

## Abstract

Magnetic and semiconducting Fe_3_O_4_/poly(o-anisidine) (POA) core/shell composite particles were fabricated by an oxidation process using Fe_3_O_4_ synthesized separately. The dispersion stability in a liquid medium and the electrical conductivity of synthesized particles were improved because of the conductive POA polymeric shell. The morphological, microstructural, compositional/elemental, and thermal behaviors of the particles were characterized using SEM with energy dispersive X-ray spectroscopy, TEM, XRD, and thermogravimetric analysis, respectively. A smart electro-magneto-rheological suspension containing Fe_3_O_4_/POA particles with two functionalities, magnetism and conductivity, was prepared. Its electrorheological properties were investigated at different electric field strengths using a rotational rheometer. Without an electric field, the sample demonstrated typical Newtonian fluid behavior, as expected. However, while under the electric field, it exhibited a solid-like behavior, and the dynamic (or elastic) yield stress of the ER fluid increased linearly as a function of the electric field strength in a power-law function with an index of 2.0, following the polarization mechanism.

## 1. Introduction

Electrorheological (ER) suspensions are smart materials that exhibit rapid changes in their rheological behaviors under an input electric field strength (*E*) [1,2], in which electro-responsive particles are dispersed in electrically insulating liquids, including mineral oil and silicone oil [3,4]. Their rheological behavior can be fine-tuned by adjusting the strength of the external electric field. Without an external *E*, they behave as Newtonian fluids [5]. However, when an *E* is applied, dipoles within the particles are driven [6,7,8]. Thus, the particles are aligned in the direction of the *E* owing to electric dipole–dipole interactions between the particles, forming a chain structure and changing its phase to a solid-like state within milliseconds [9,10,11]. This behavior is reversible when the *E* is removed again, wherein it returns to the liquid state and exhibits Newtonian fluid-like behavior [12,13,14,15,16,17]. Due to their artificial controllability and drastic responses and excellent mechanical properties with high yield stresses, ER suspensions have been adopted in various industrial fields such as clutches, brakes, dampers, and shock absorbers [18,19].

Among the various potential electro-responsive materials for ER suspensions, conducting polymers, such as polyaniline (PANI), polydiphenylamine (PDPA), and polypyrrole (PPy) [20,21,22,23], have been widely adopted because of their controllable electrical conductivity and dielectric properties. In addition, conductive polymer/inorganic composite particles with various shapes have been introduced to improve ER performance. In order to minimize the energy generated by their large surface volume ratio, nanoparticles tend to form aggregates, leading to a decrease in the efficiency in the ER fluids. However, by preparing the polymeric shell structure around the particles, the nanoparticles cannot be agglomerated and precipitated, keeping the dispersion stability for a long time [24,25,26]. In particular, particles with a variety of core–shell structured-combinations, including conductive shell/dielectric cores or conductive shell/magnetic cores, are widely used owing to their synergetic combination of the respective functional properties provided from the core and shell [27,28,29,30].

In contrast, soft-magnetic carbonyl iron (CI) spheres, used as cores of composite particles, have been widely applied to magnetorheological (MR) fluids because of their excellent controllable magnetic properties and suitable and tunable particle size [31,32,33]. Although CI particles have several such advantages, CI particle-based MR fluids face problems of sedimentation and re-dispersion owing to their high density (approximately 8.0 g/cm^3^), which limits their engineering applications [34,35,36]. In order to overcome these problems, magnetite (Fe_3_O_4_) particles with relatively low density and sufficient magnetic responses can be selected. However, there are still problems which need to be improved, such as the Fe^2+^ in Fe_3_O_4_ particles [37,38] being easily oxidized or corroded in the air. Covering the surface of Fe_3_O_4_ particles with organic polymers could help overcoming the problem of Fe^2+^ being oxidized [39,40], and make Fe_3_O_4_ particles have better dispersion stability in ER fluids [41,42]. In particular, coating with conducting polymers can produce composite particles with conductive shell/magnetic core structures that can be used not only for MR, but also for ER suspensions [43,44].

**Scheme 1:**
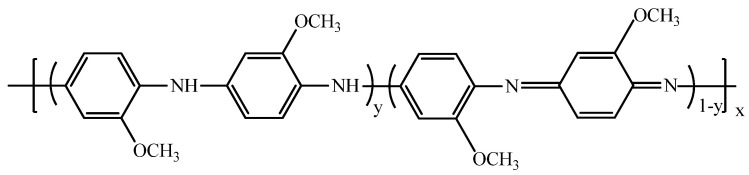
Chemical structure of poly(o-anisidine).

Polyaniline (PANI) is an attractive conducting polymer because of its higher corrosion resistance compared to other conducting polymers and its easily tunable conductivity [45]. However, its application is severely limited by its poor solubility, workability, and adhesion between the material and metal materials and pinhole defects [46,47]. The introduction of alkyl, alkoxy, amine, and imine groups on the benzene ring can significantly enhance the physical characteristics and anti-corrosion behavior of PANI, possibly due to both steric restrictions and phi-electron effects [48]. In addition, the increased molecular size of the substituted PANI can facilitate better surface coverage and lead to better adhesion of the metal substrate. One of the PANI derivatives, poly(o-anisidine) (POA), whose chemical structure is shown in Scheme 1, is soluble in various solvents because the methoxy group at the benzene ring of POA is connected to the amino group, which provides good solution processing properties. Related to its electrical conductivity, in particular, it can be directly applied to ER fluids owing to its semiconducting properties.

Here, we prepared Fe_3_O_4_/POA (core/shell) composite particles by coating the synthesized Fe_3_O_4_ particles with POA, which has high reactivity and easily controllable conductivity, and fabricated a suspension with composite particles. Fe_3_O_4_/POA composite particles possessing a combination of dielectricity and magnetism particles showed dual stimuli responses. It can be further noted that our Fe_3_O_4_/POA (core/shell) composite particles possess advantages compared to previously reported Fe_3_O_4_/PANI [41] and Fe_3_O_4_/TiO_2_ [49] (core/shell) composite particles in which the PANI needs to be de-doped for ER measurements, and inorganic TiO_2_ might not provide a better dispersibility than POA in terms of its density. The morphologies and structures, sizes, compositions, and thermal stabilities of the Fe_3_O_4_/POA composite particles were tested using scanning electron microscopy (SEM), transmission electron microscopy (TEM), X-ray diffraction (XRD), and thermogravimetric analysis (TGA). We discuss the ER behavior of the fabricated suspensions with the composite particles under various *E*s using a rotational rheometer in this paper, in addition to the MR characteristics demonstrated in a previous publication [50].

## 2. Experimental

### 2.1. Preparation of Fe_3_O_4_ and Fe_3_O_4_/POA Core–Shell Particles

The synthesis of Fe_3_O_4_ particles and the fabrication process of Fe_3_O_4_/POA core–shell composite particles were described in detail in our previous publication [50]. Fe_3_O_4_ particles were synthesized using a solvothermal process using a Teflon-coated stainless-steel autoclave. The resulting Fe_3_O_4_ particles were then placed in a reactor containing 0.1 M HCl under stirring for 10 h at 5 °C. Then, both ethanol (100 mL, DUCKSAN, Gwangju-si, Korea) and ortho-anisidine monomer (1 mL, DUCKSAN, Gwangju-si, Korea) were added to the reactor in a nitrogen atmosphere. After 8 h, 12 M HCl and ammonium persulfate were added dropwise to the mixture for 30 min. The resulting Fe_3_O_4_ particles coated with POA were then cleaned with ethyl alcohol and distilled water several times and dried in an oven for 1 day.

Finally, an ER suspension with 10 vol% of the prepared Fe_3_O_4_/POA composite particles was fabricated and dispersed uniformly in silicone oil with a viscosity of 100 cSt.

### 2.2. Characterization

The morphology and elemental component of the Fe_3_O_4_/POA composite particles were characterized by high-resolution SEM ((HR-SEM, SU-8010, Hitachi, Tokyo, Japan) at 15 kV, 8.0 mm × 120 k (magnification) SE (U) coupled with energy-dispersive X-ray spectroscopy (EDS) peaks ranging from 0 to 8 keV (EX-250, HORIBA, Kyoto, Japan) at 15 kV, 15.0 mm × 2 k SE (U). TEM (CM-220, Phillips, Amsterdam, The Netherlands) was used to observe the core–shell structure of the composite particles, while XRD (DMax-2500, Rigaku, Tokyo, Japan, 2θ range from 10 to 80 degree) analysis was used to study the crystal structures of the fabricated particles. The thermal stabilities of the Fe_3_O_4_ and POA/Fe_3_O_4_ particles were further measured and compared with those of pure POA molecules using TGA (TG 209 F3, NETZSCH, Gebrüder, Germany) by heating to 800 °C (heating rate: 10 °C/min) in nitrogen environment. The ER characteristics of the smart suspension with the prepared Fe_3_O_4_/POA composite particles were obtained using a rotational rheometer (MCR300, Anton Paar, Graz, Austria) with an electric field from 0 to 2 kV/mm, which was connected to a high-voltage supplier (HCN 7E-12500, FuG Elektronik, Germany) along with a concentric cylinder mode (CC 17/E).

## 3. Results and Discussion

### 3.1. Material Properties

**Figure 1:**
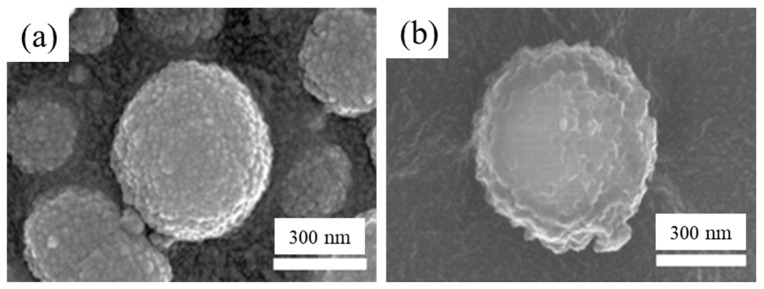
SEM photos of (**a**) the pure Fe_3_O_4_ particles and (**b**) the Fe_3_O_4_/POA core–shell composite particles.

**Figure 2:**
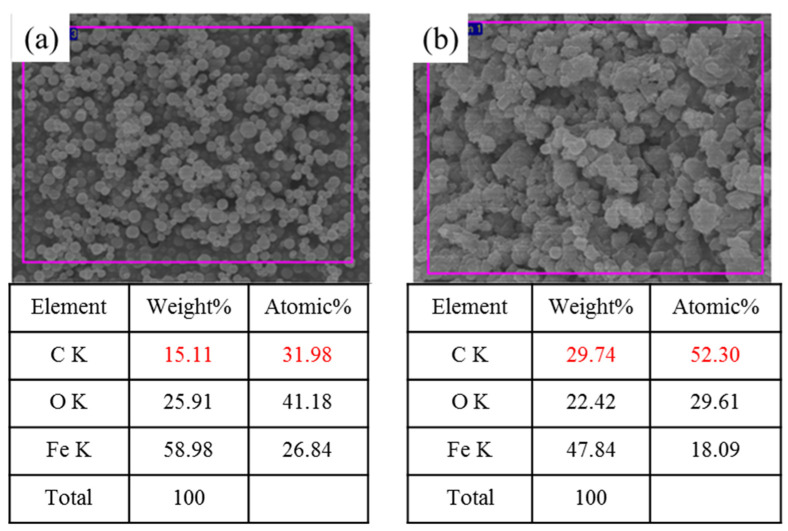
SEM images and EDS data of (**a**) the pure Fe_3_O_4_ particles and (**b**) the Fe_3_O_4_/POA core–shell composite particles. Tables show the element information of the EDS spectra of the selected parts noted as pink squares in the corresponding SEM images for both types.

Figure 1 and Figure 2 present the SEM images of the shape and size of the pure Fe_3_O_4_ and Fe_3_O_4_/POA particles fabricated in this work. It is evident that the Fe_3_O_4_ particles have a spherical shape and rough surface. In addition, their size is approximately 300 nm, and spherical POA-coated Fe_3_O_4_ composite particles also have a rough surface. Both particles have a multi-dispersed size distribution [49], and their slight aggregation was examined. Tables shown in Figure 2a,b present the elemental information of the EDS spectra of the Fe_3_O_4_ and Fe_3_O_4_/POA particles in the selected part, indicated as pink squares in the corresponding SEM images. The Fe_3_O_4_ particles contained Fe (58.98%), O (25.91%), and C (15.11%), and the core–shell-structured Fe_3_O_4_/ POA composite particles were composed of Fe (47.84%), O (22.43%), and C (29.74 %). The tables indicate that the carbon content increased from 15.11% to 29.74%, due to the shell portion formed by coating the pure Fe_3_O_4_ particles with POA. 

**Figure 3:**
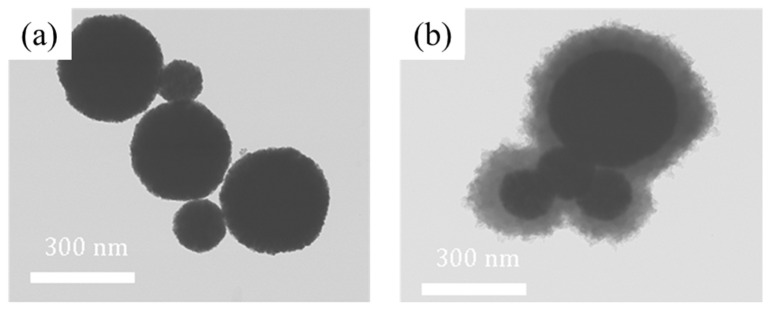
TEM photos of (**a**) the Fe_3_O_4_ particles and (**b**) the Fe_3_O_4_/POA composite particles.

Figure 3 display the TEM images of the fabricated Fe_3_O_4_ and Fe_3_O_4_/POA composite particles, respectively. It can be observed that the Fe_3_O_4_/POA particles are in the core–shell type that was most probably induced by absorbing ortho-anisidine monomers on the surface of the Fe_3_O_4_ particles through hydrogen bonding and electrostatic attraction after acidification of the Fe_3_O_4_ spheres with HCl. The surface of the Fe_3_O_4_ particles was modified only through the acidification step, and no other surface treatments were necessary. The average diameter of the Fe_3_O_4_ particles was estimated to be roughly 300 nm, and the mean thickness of the POA shell was around 60 nm. The observed core–shell form of the composite particles indicates that the Fe_3_O_4_ particles were successfully coated with the conducting POA molecules and exhibited electric and magnetic properties concurrently. Furthermore, an FT-IR analysis of the POA-coated spheres confirming their synthesis can be found in our previous report [50].

**Figure 4:**
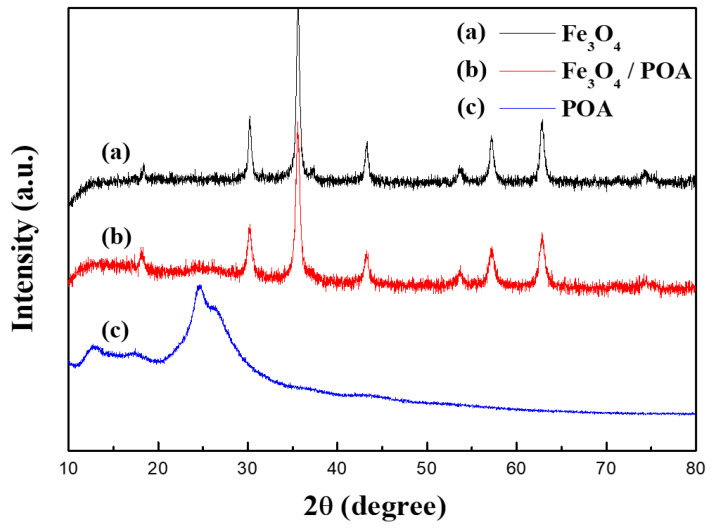
XRD data of (**a**) pure Fe_3_O_4_, (**b**) the Fe_3_O_4_/POA composite particles, and (**c**) the POA particles.

Figure 4 shows an XRD pattern between 10° and 80° for the POA molecules and Fe_3_O_4_/POA and pure Fe_3_O_4_ particles. The peaks in the pattern (Figure 4a) for the pure conducting POA molecules are also similar to those of other typical polymers exhibiting broad peaks at low angles. The pattern of the Fe_3_O_4_/POA composite particles (Figure 4b) showed more amorphous peaks in the low 2θ range (20–30° 2θ) due to the POA shell part compared to the pure Fe_3_O_4_ particles, for which the peaks of the crystal structure at 2θ were 18.31°, 30.11°, 36.51°, 43.21°, 53.51°, 57.11°, and 62.61° [51] (Figure 4c).

**Figure 5:**
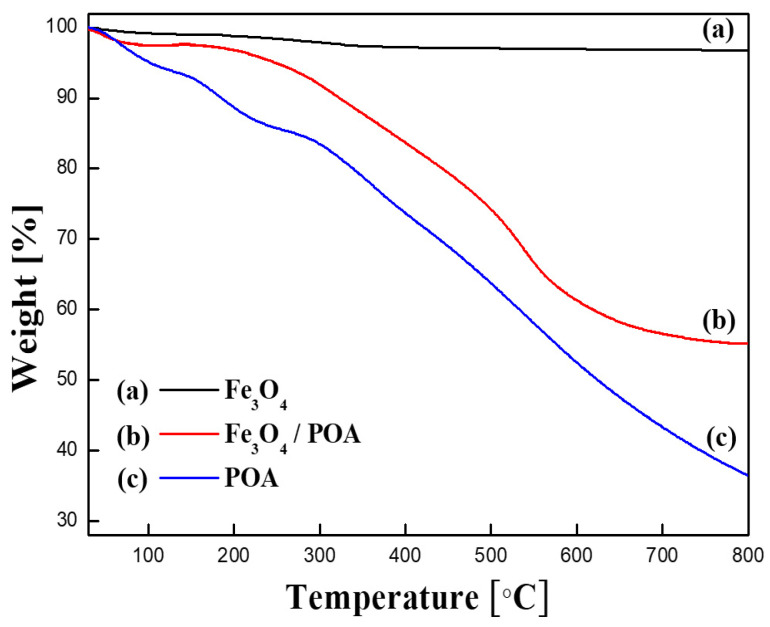
TGA diagram of the (**a**) pure Fe_3_O_4_, (**b**) Fe_3_O_4_/POA particles and (**c**) POA.

When the Fe_3_O_4_/POA, pure Fe_3_O_4_ particles, and pure POA were heated under a 99% nitrogen environment, their weight changed with temperature, as shown in Figure 5. The TGA curve of the fabricated Fe_3_O_4_ particles showed a slight weight loss over the entire temperature range, as expected (Figure 5a). The weight loss at approximately 100 °C was caused by moisture evaporation. Above that, the decomposition of the POA steadily reduced the weight up to 600 °C and 800 °C for Fe_3_O_4_/POA and Fe_3_O_4_ particles, respectively, owing to the large-scale thermal decomposition of the POA chains [52] (Figure 5b,c). Moreover, at temperatures up to 800 °C, 63.5% of the POA and 44.8% of the Fe_3_O_4_/POA composite particles were decomposed. The interaction between the Fe_3_O_4_ particles and the POA chains probably limited the thermal motion of the Fe_3_O_4_ particles and could provide thermal stability to the composite particles. These results also indicate that the magnetic properties of the coated Fe_3_O_4_/POA composite particles decreased due to the decrease in saturation magnetization resulting from the addition of a non-magnetic POA shell. Their densities, measured using a gas pycnometer (AccuPyc 1130), also decreased, from 4.34 g/cm^3^ to 2.52 g/cm^3^ [50].

### 3.2. Electrorheological (ER) Characteristics

**Figure 6:**
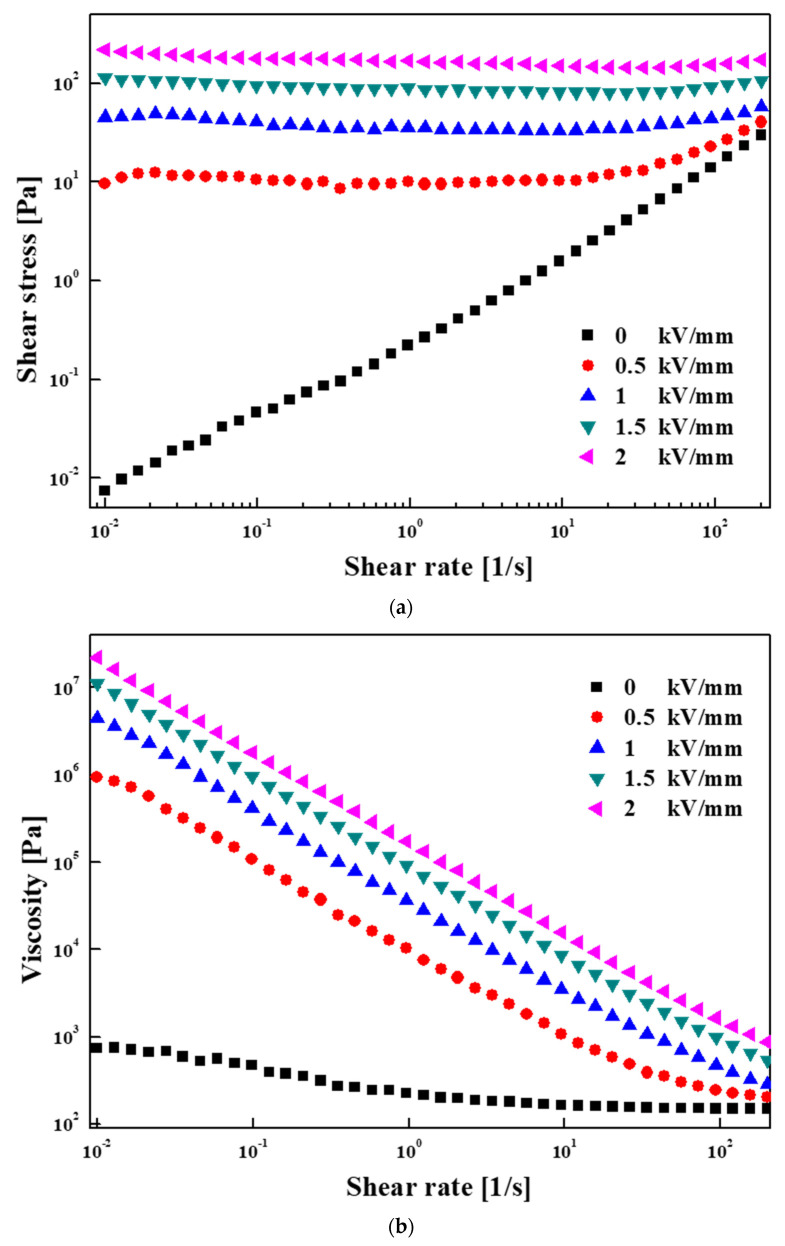
(**a**) Shear stress and (**b**) shear viscosity for core–shell Fe_3_O_4_/POA particle (10 vol%)-based ER suspension under different *E*s.

The ER behavior of the suspension with the 10 vol% Fe_3_O_4_/POA composite particles suspended in silicone oil (100 cSt at 25 °C) under various electric fields was scrutinized using a rheometer equipped with a concentric cylinder (CC) mode at room temperature. Constant steady shear flow tests of the Fe_3_O_4_/POA-based ER suspension were performed as the *E* increased from 0 to 2 kV/mm. The shear stress (*τ*) and shear viscosity of the ER fluid are shown as a function of the shear rate in Figure 6a,b, respectively. Without an electric field, its viscosity over the shear rate was shown to change slightly, and its shear stress increased linearly with increased shear rate. These results indicate that the Fe_3_O_4_/POA-based ER suspension behaved as a Newtonian fluid without an applied external *E*. In addition, the shear viscosity of the overall suspension was slightly higher than that of pure silicone oil because of the addition of 10 vol% Fe_3_O_4_/POA particles. Meanwhile, when an *E* was applied, the suspension showed both a solid-like behavior with a yield stress and the shear stress with a flat region over the entire shear rate. The yield stress, which is the minimum shear stress needed to break a particle chain-like structure formed under an electric field applied between cylinders, viscosity, and the flat region of shear stress of the suspension increased with increasing the applied *E*. As for the shear viscosity of the sample suspension, due to the application of the electric field, the dispersed particles in the fluid formed a chain-like structure; thus, the overall viscosity increased with the increase in the electric field.

**Figure 7:**
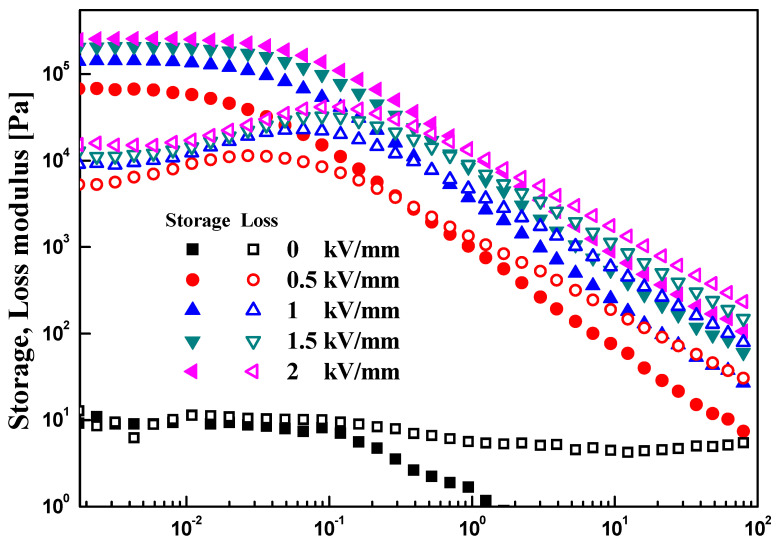
*G*′ (closed) and *G*″ (open) for the core–shell Fe_3_O_4_/POA particle-based ER suspension under different *E*s as a function of strain.

**Figure 8:**
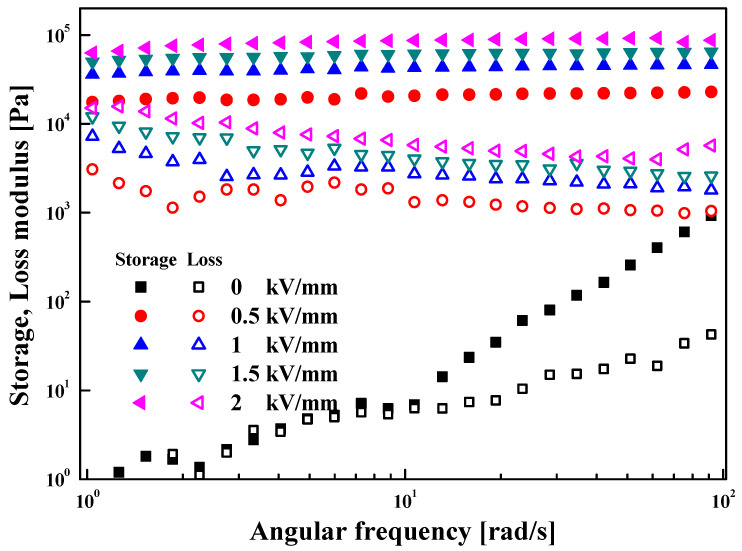
*G*′ (closed) and *G*″ (open) for the core–shell Fe_3_O_4_/POA particle-based ER suspension under various *E*s as a function of angular frequency.

The viscoelastic behavior of the Fe_3_O_4_/POA-based ER suspension was analyzed by measuring the storage modulus (*G*′) and loss modulus (*G*″) through dynamic oscillatory tests. The evaluation of the behaviors of ER fluids at sufficiently low oscillating strain amplitudes has been established as an adequate method to examine their dynamic deformation behaviors when their structures are not destroyed in the linear viscoelastic region (LVE). Figure 7 shows the *G*′ and *G*″ of the ER suspension at a fixed angular frequency of 1 Hz as a function of strain. The *G*′ and *G*″ of the suspension in the absence of an *E* exhibited similar values in the initial shear strain range. However, under an applied *E*, the *G*′ was much larger than the *G*″. This implies that a strong phase transition in the suspension occurred under an applied *E*. The LVE of the suspension widened with increasing electric field strength. This indicated the solidification ability of the EP particles. When the strain exceeded the LVE, its storage modulus decreased because the chain-like microstructure of the ER suspension was deformed and broken, decreasing the *G*′ and *G*″; this is known as strain softening, also called the Payne effect [53,54]. In the high strain region, its *G*″ was larger than its *G*′, which meant that the ER suspension exhibited viscoelastic fluid properties. A strain of 0.004% from the LVE was selected for frequency sweep tests of the suspension, and at a constant strain of 0.004%, the *G*′ and *G*″ values of the Fe_3_O_4_/POA-based suspension under four different *E*s were determined as functions of angular frequency (see Figure 8). Its *G*′ values were higher than the *G*″ values over the whole angular frequency range, indicating that the elastic behavior of the ER suspension dominated its viscous behavior. In addition, both moduli almost showed a plateau and increased with increasing *E* over the entire frequency range, which indicated that the elastic and viscous properties of the ER suspension increased when a higher *E* was applied. In other words, the stronger the *E* produced, the stronger the chain-like structure and damping property of the ER fluid.

**Figure 9:**
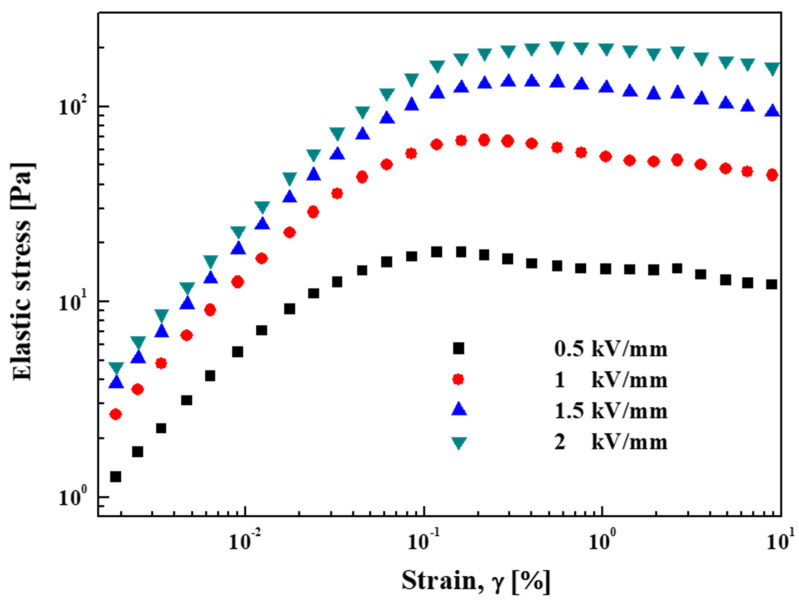
Elastic stress of the core–shell Fe_3_O_4_/POA particle (10 vol%)-based ER suspension as a function of strain.

Figure 9 presents the elastic stress, $\tau^{\prime }$, obtained through a subsequent analysis using the relationship $\tau^{\prime }=G^{\prime }\cdot \;\gamma$, where γ is the strain amplitude of the suspension under various *E*s (from 0.5 to 2.0 kV/mm). The elastic yield stress was determined from the point where the $\tau^{\prime }$ approached the highest stress value just before decreasing because the chain-like structure formed in the ER suspension began to be destroyed; this point is indicated by a red circle for each curve.

**Figure 10:**
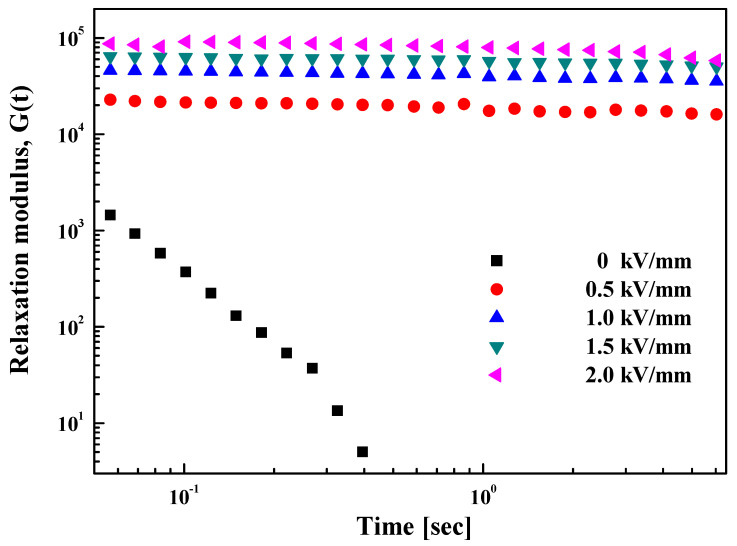
Relaxation modulus, *G(t),* of the core–shell particle-based ER suspension, obtained by the storage and loss modulus obtained from a frequency sweep test and using Equation (2), as a function of time.

Figure 10 demonstrates the time-dependent relaxation shear modulus, *G(t)*, of the Fe_3_O_4_/POA-based ER suspension determined from its *G*′ and *G*″ as a function of frequency (*G*′(*ω*) and *G*″(*ω*), respectively) using the Schwarzl equation (Equation (1)) [55]. The *G(t)* of the ER suspension under an *E* showed a plateau over time, due to the solid-like behavior of the suspension, while *G(t)* decreased linearly because of its fluid-like behavior without an *E*.

(1) $$G\left(t\right)=G^{\prime }\left(\omega \right)-0.566G^{\prime \prime }\left(\omega \right)+0.203G^{\prime \prime }\left(\omega \right){|}_{\omega =1/t}$$

**Figure 11:**
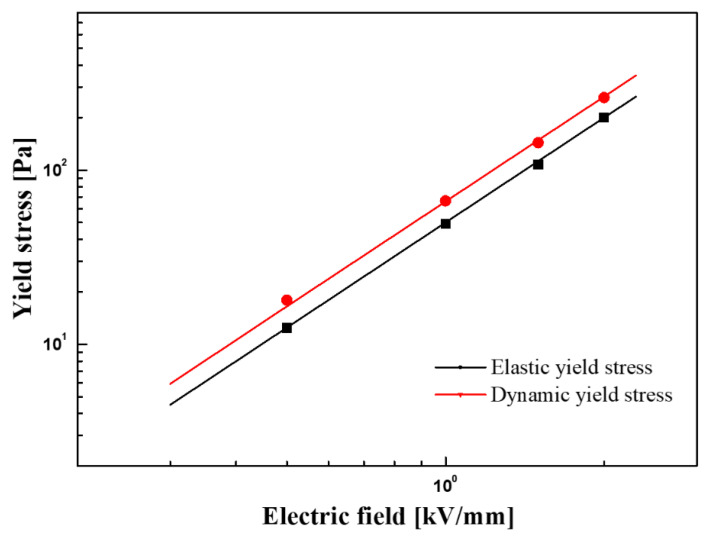
Dynamic and elastic yield stresses of the core–shell particle (10 vol%)-based ER suspension as a function of an electric field.

The dynamic yield stress, which was determined by extrapolating the τ to the zero-limit shear rate in the flow curve (see Figure 6), and the elastic yield stress determined from the red circle in the elastic stress curve (see Figure 9) for the Fe_3_O_4_/POA-based ER fluid, are shown as a function of *E* in Figure 11. When an *E* was applied, the plot of yield stress *τ_y_* versus the electric field *E* typically showed an exponential relationship as follows:

(2) $$\tau_{y}\propto E^{\alpha }$$

In general, the power-law index is 1.5 for a conduction mechanism and 2.0 for a polarization mechanism. The index for the Fe_3_O_4_/POA-based ER suspension was determined to be approximately 2, which indicated that the suspension followed the polarization model [23,56,57].

## 4. Conclusions

New core–shell-type Fe_3_O_4_/POA composite particles were fabricated by coating Fe_3_O_4_ particles with a conductive POA to enhance the dispersion stability of the particles and provide electrical conductivity to the particles based on our previously published results [50]. The Fe_3_O_4_ particles were synthesized via a hydrothermal method, and the core–shell-structured Fe_3_O_4_/POA composite particles were fabricated via the in situ oxidation polymerization of POA onto the surface of Fe_3_O_4_ particles. The POA shell not only effectively prevented the aggregation tendency of internal Fe_3_O_4_ particles, but also protected the internal particles from external oxidation and prevented changes in the original structure that might have occurred in the presence of other external magnetic fields. Their successful production of the core–shell form was confirmed by SEM and TEM analyses. The core size of the Fe_3_O_4_ particles and shell thickness of the POA in the spherical composite core/shell particles were about 300 nm and 60 nm, respectively. This suitable and small particle size not only improved the stability of ER suspension, but also improved the ER effect when the electric field was applied.

The ER properties of the suspension prepared by dispersing the composite particles in an insulating silicone oil were studied using a rheometer for various electric field strengths. The *G*′ and *G*″ of the ER suspension displayed typical Newtonian fluid characteristics without an *E*, and increased with increasing *E*. The relaxation modulus obtained using the Schwarzl equation clearly showed a solid-like behavior under applied electric fields, compared to a liquid-like behavior without an *E*. Furthermore, the suspension also exhibited a yield stress because the higher electric field induced a more robust, chain-like structure. The apparent yield stress of the suspension was determined to be approximately 200 Pa at 2 kV/mm, and both dynamic and elastic yield stresses increased linearly with the *E* in a power-law function with a slope of 2.0, following the polarization mechanism.

The findings of this study suggest that a smart suspension containing Fe_3_O_4_/POA composite particles composed of a conducting shell and magnetic core under an applied external field exhibits significant ER properties. In addition, this Fe_3_O_4_/POA composite has a great possibility to be applied in various fields including brakes, dampers, elastomers, flexible tactile sensors, and advanced medical instruments, because of the high yield stress under weak electric fields.

## Data Availability

The data presented in this study are available on request from the corresponding author.

## References

[1] Kuznetsov N.M., Zagoskin Y.D., Vdovichenko A.Y., Bakirov A.V., Kamyshinsky R.A., Istomina A.P., Grigoriev T.E., Chvalun S.N. (2021). Enhanced electrorheological activity of porous chitosan particles. Carbohydr. Polym..

[2] Bica I., Anitas E.M., Averis L.M.E. (2015). Tensions and deformations in composites based on polyurethane elastomer and magnetorheological suspension: Effects of the magnetic field. J. Ind. Eng. Chem..

[3] Shen M., Huang Q. (2016). Acoustic velocity and attenuation coefficient of magnetorheological fluids under electromagnetic fields. Appl. Acoust..

[4] Cvek M., Mrlik M., Ilcikova M., Plachy T., Sedlacik M., Mosnacek J., Pavlinek V. (2015). A facile controllable coating of carbonyl iron particles with poly(glycidyl methacrylate): A tool for adjusting MR response and stability properties. J. Mater. Chem. C.

[5] Yin J., Zhao X. (2011). Electrorheology of nanofiber suspensions. Nanoscale Res. Lett..

[6] Plachy T., Sedlacik M., Pavlinek V., Stejskal J. (2015). The observation of a conductivity threshold on the electrorheological effect of p-phenylenediamine oxidized with p-benzoquinone. J. Mater. Chem. C.

[7] Zheng C., Liu Y., Dong Y., He F., Zhao X., Yin J. (2019). Low-Temperature Interfacial Polymerization and Enhanced Electro-Responsive Characteristic of Poly(ionic liquid)s@polyaniline Core-shell Microspheres. Macromol. Rapid Commun..

[8] Kutalkova E., Plachy T., Sedlacik M. (2020). On the enhanced sedimentation stability and electrorheological performance of intelligent fluids based on sepiolite particles. J. Mol. Liq..

[9] Cho Y.H., Cho M.S., Choi H.J., Jhon M.S. (2002). Electrorheological characterization of polyaniline-coated poly(methyl methacrylate) suspensions. Colloid Polym. Sci..

[10] Chen P., Cheng Q., Wang L.-M., Liu Y.D., Choi H.J. (2019). Fabrication of dual-coated graphene oxide nanosheets by polypyrrole and poly(ionic liquid) and their enhanced electrorheological responses. J. Ind. Eng. Chem..

[11] Do T., Lee H., Ko Y.G., Chun Y., Choi U.S., Kim C.H. (2017). Influence of amine- and sulfonate-functional groups on electrorheological behavior of polyacrylonitrile dispersed suspension. Colloids Surf. A.

[12] Kuznetsov N.M., Belousov S.I., Kamyshinsky R.A., Vasiliev A.L., Chvalun S.N., Yudina E.B., Vul A.Y. (2021). Detonation nanodiamonds dispersed in polydimethylsiloxane as a novel electrorheological fluid: Effect of nanodiamonds surface. Carbon.

[13] Plachy T., Masar M., Mrlik M., Machovsky M., Machovska Z., Kutalkova E., Kuritka I. (2019). Switching between negative and positive electrorheological effect of g-C_3_N_4_ by copper ions doping. Adv. Powder Technol..

[14] Xi Z., Ma J., Sun W., Wang B., Hao C. (2020). Synthesis and electrorheological properties of hierarchical and core-shell MoS_2_@ TiO_2_ nanocomposite. J. Solid State Chem..

[15] Zhao J., Lei Q., He F., Zheng C., Zhao X., Yi J. (2020). Influence of geometry of mobile countercations on conductivity, polarization and electrorheological effect of polymeric anionic liquids at ice point temperature. Polymer.

[16] Gercek B., Yavuz M., Yilmaz H., Sari B., Unal H.I. (2007). Comparison of electrorheological properties of some polyaniline derivatives. Colloids Surf. A.

[17] Gao C.Y., Kim M.H., Jin H.-J., Choi H.J. (2020). Synthesis and Electrorheological Response of Graphene Oxide/Polydiphenylamine Microsheet Composite Particles. Polymers.

[18] Ma N., Yao Y., Wang Q., Niu C., Dong X. (2020). Properties and mechanical model of a stiffness tunable viscoelastic damper based on electrorheological elastomers. Smart Mater. Struct..

[19] Chiolerio A., Quadrelli M.B. (2017). Smart fluid systems: The advent of autonomous liquid robotics. Adv. Sci..

[20] Stejskal J., Mrlík M., Plachý T., Trchová M., Kovářová J., Li Y. (2017). Molybdenum and tungsten disulfides surface-modified with a conducting polymer, polyaniline, for application in electrorheology. React. Funct. Polym..

[21] Tian X., He K., Wang C., Wen Q., Wang B., Yu S., Hao C., Chen K., Lei Q. (2016). Preparation and electrorheological behavior of anisotropic titanium oxide/polyaniline core/shell nanocomposite. Compos. Sci. Technol..

[22] Kim M.H., Bae D.H., Choi H.J., Seo Y. (2017). Synthesis of semiconducting poly(diphenylamine) particles and analysis of their electrorheological properties. Polymer.

[23] Kim Y.D., Yoon D.J. (2016). Electrorheological fluids of polypyrrole-tin oxide nanocomposite particles. Korea-Aust. Rheol. J..

[24] Cabuk S., Unal H.I. (2015). Enhanced electrokinetic, dielectric and electrorheological properties of covalently bonded particles-TiO_2_/polypyrrole nanocomposite. React. Funct. Polym..

[25] Zhu X., Zhao Q., Zhang T., Pang X. (2019). Electrorheological Response of Novel polyaniline-Fe_2_O_3_ Nanocomposite Particles. Polym. Plast. Tech. Mater..

[26] Erol O., Unal H.I. (2015). Core/shell-structured, covalently bonded TiO_2_/poly(3,4-ethylenedioxythiophene) dispersions and their electrorheological response: The effect of anisotropy. RSC Adv..

[27] Tian X., He K., Wang B., Yu S., Hao C., Chen K., Lei Q. (2016). Flower-like Fe_2_O_3_/polyaniline core/shell nanocomposite and its electroheological properties. Colloids Surf. A.

[28] Piao S.H., Gao C.Y., Choi H.J. (2017). Sulfonated polystyrene particles coated with conducting polyaniline and their electro-responsive suspension characteristics under electric fields. Polymer.

[29] Wang X., Qian X., Jiang X., Lu Z., Hou L. (2016). Tunable electrorheological characteristics and mechanism of a series of graphene-like molybdenum disulfide coated core–shell structured polystyrene microspheres. RSC Adv..

[30] Dong Y.Z., Choi H.J. (2018). Synthesis of organic–inorganic poly(diphenylamine)/magnetite composite particles and their magnetorheological response. IEEE Trans. Magn..

[31] Mrlík M., Ilčíková M., Pavlínek V., Mosnáček J., Peer P., Filip P. (2013). Improved thermooxidation and sedimentation stability of covalently-coated carbonyl iron particles with cholesteryl groups and their influence on magnetorheology. J. Colloid Interface Sci..

[32] Machovsky M., Mrlik M., Kuritka I., Pavlinek V., Babayan V. (2014). Novel synthesis of core–shell urchin-like ZnO coated carbonyl iron microparticles and their magnetorheological activity. RSC Adv..

[33] Plachy T., Kutalkova E., Sedlacik M., Vesel A., Masar M., Kuritka I. (2018). Impact of corrosion process of carbonyl iron particles on magnetorheological behavior of their suspensions. J. Ind. Eng. Chem..

[34] Hong C.H., Kim M.W., Zhang W.L., Moon I.J., Choi H.J. (2016). Fabrication of smart magnetite/reduced graphene oxide composite particles and their magnetic stimuli-response. J. Colloid Interface Sci..

[35] Wang G., Ma Y., Cui G., Li N., Dong X. (2018). Two-dimensional Fe_3_O_4_/MoS_2_ nanocomposites for a magnetorheological fluid with enhanced sedimentation stability. Soft Matter.

[36] Plachy T., Cvek M., Kozakova Z., Sedlacik M., Moucka R. (2017). The enhanced MR performance of dimorphic MR suspensions containing either magnetic rods or their non-magnetic analogs. Smart Mater. Struct..

[37] Ruan X., Pei L., Xuan S., Yan Q., Gong X. (2017). The rheological responds of the superparamagnetic fluid based on Fe_3_O_4_ hollow particles. J. Magn. Magn. Mater..

[38] Chae H.S., Kim S.D., Piao S.H., Choi H.J. (2016). Core-shell structured Fe_3_O_4_@ SiO_2_ particles fabricated by sol–gel method and their magnetorheology. Colloid Polym. Sci..

[39] Guo Z., Henry L.L., Palshin V., Podlaha E.J. (2006). Synthesis of poly(methyl methacrylate) stabilized colloidal zero-valence metallic particles. J. Mater. Chem..

[40] Jiang W., Zhu H., Guo C., Li J., Xue Q., Feng J., Gong X. (2010). Poly(methyl methacrylate)-coated carbonyl iron particles and their magnetorheological characteristics. Polym. Int..

[41] Noh J., Hong S., Yoon C.-M., Lee S., Jang J. (2017). Dual external field-responsive polyaniline-coated magnetite/silica particles for smart fluid applications. Chem. Commun..

[42] Dong Y.Z., Choi H.J. (2018). Synthesis of Smart Poly(diphenylamine)/Magnetic Particle Composites and Their Electric/Magnetic Stimuli-Response. Macromol. Res..

[43] Hsieh T.-H., Ho K.-S., Bi X., Han Y.-K., Chen Z.-L., Hsu C.-H., Chang Y.-C. (2009). Synthesis and electromagnetic properties of polyaniline-coated silica/maghemite particles. Eur. Polym. J..

[44] Dong Y.Z., Esmaeilnezhad E., Choi H.J. (2021). Core–Shell Structured Magnetite-Poly(diphenylamine) Microspheres and Their Tunable Dual Response under Magnetic and Electric Fields. Langmuir.

[45] Lee J.H., Cho M.S., Choi H.J., Jhon M.S. (1999). Effect of polymerization temperature on polyaniline based electrorheological suspensions. Colloid Polym. Sci..

[46] Abaci S., Nessark B. (2015). Characterization and corrosion protection properties of composite material (PANI+TiO_2_) coatings on A304 stainless steel. J. Coat. Technol. Res..

[47] Sazou D., Deshpande P.P. (2017). Conducting polyaniline nanocomposite-based paints for corrosion protection of steel. Chem. Pap..

[48] Andriianova A.N., Biglova Y.N., Mustafin A.G. (2020). Effect of structural factors on the physicochemical properties of functionalized polyanilines. RSC Adv..

[49] Yin Y., Liu C., Wang B., Yu S., Chen K. (2013). The synthesis and properties of bifunctional and intelligent Fe_3_O_4_@titanium oxide core/shell nanoparticles. Dalton Trans..

[50] Lee J.H., Lu Q., Lee J.Y., Choi H.J. (2019). Polymer-Magnetic Composite Particles of Fe_3_O_4_/Poly(o-anisidine) and Their Suspension Characteristics under Applied Magnetic Fields. Polymers.

[51] Deshpande P.P., Murali M., Deshpande P.P., Galphade V.S., More M.A. (2013). Conducting poly(o-anisidine)-coated steel electrodes for supercapacitors. Chem. Pap..

[52] Khan A.A., Shaheen S., Habiba U. (2012). Synthesis and characterization of poly-o-anisidine Sn(IV) tungstate: A new and novel ‘organic–inorganic’ nano-composite material and its electro-analytical applications as Hg(II) ion-selective membrane electrode. J. Adv. Res..

[53] Fang F.F., Choi H.J., Jhon M.S. (2009). Magnetorheology of soft magnetic carbonyl iron suspension with single-walled carbon nanotube additive and its yield stress scaling function. Colloids Surf. A.

[54] Cabuk M. (2017). Electrorheological response of mesoporous expanded perlite particles. Microporous Mesoporous Mater..

[55] Schwarzl F. (1975). Numerical calculation of stress relaxation modulus from dynamic data for linear viscoelastic materials. Rheol. Acta.

[56] Stenicka M., Pavlinek V., Saha P., Blinova N.V., Stejskal J., Quadrat O. (2009). The electrorheological efficiency of polyaniline par ticles with various conductivities suspended in silicone oil. Colloid Polym. Sci..

[57] Jang W.H., Kim J.W., Choi H.J., Jhon M.S. (2001). Synthesis and electrorheology of camphorsulfonic acid doped polyaniline suspensions. Colloid Polym. Sci..

